# Small fallopian tube carcinoma with extensive upper abdominal dissemination: a case report

**DOI:** 10.1186/1752-1947-7-252

**Published:** 2013-11-07

**Authors:** Carolina Oliveira, Hálio Duarte, Carla Bartosch, Daniel Fernandes

**Affiliations:** 1 Oncological Gynecology Department, Instituto Português de Oncologia do Porto, Rua Dr. António Bernardino de Almeida, 4200-072 Porto, Portugal; 2 Radiology Department, Instituto Português de Oncologia do Porto, Rua Dr. António Bernardino de Almeida, 4200-072 Porto, Portugal; 3 Pathology Department, Instituto Português de Oncologia do Porto, Rua Dr. António Bernardino de Almeida, 4200-072 Porto, Portugal; 4 Permanent address: Oncological Gynecology Department, Instituto Português de Oncologia do Porto, Avenida D. Manuel II, 1388, 6º andar, 4470-334 Maia, Portugal

**Keywords:** Fallopian tube carcinoma, Histology, Prognostic factors, Review, Symptoms

## Abstract

**Introduction:**

Fallopian tube carcinoma is a rare gynecological malignancy with low accuracy detection preoperatively. The symptoms are unspecific and imaging can be misleading. Since it was first described in 1847, there have been only a little over 2000 case reports.

**Case presentation:**

This case report describes a 66-year-old Caucasian woman who presented with progressive diffuse abdominal pain, without other symptoms. After abdominopelvic magnetic resonance imaging, she was sent to the Portuguese Oncology Institute of Oporto with the suspicion of peritoneal carcinomatosis of unknown primary tumor. Due to a pelvic palpable mass (calcified giant uterine fibroid) she was directed to the Gynecology team. Surgery was performed and a large mass in her upper abdomen was identified. The extemporary examination revealed a high-grade adenocarcinoma. During surgery a small change of color and consistency of her left fallopian tube was noted and unilateral adnexectomy was performed. After pathologic and immunohistochemistry tests, the diagnosis of fallopian tube carcinoma with peritoneal dissemination was made.

**Conclusions:**

This case is very unique in the way that a small primary fallopian tube carcinoma was able to disseminate to the upper abdominal quadrant with little pelvic dissemination. The symptoms and imaging were unspecific. Although a rare occurrence, we should not forget fallopian tube carcinoma in the differential diagnosis of peritoneal carcinomatosis, even in the absence of Latzke’s triad.

## Introduction

Primary fallopian tube carcinoma (PFTC) is a rare gynecological malignancy that accounts for 0.14 to 1.8% of all gynecological cancers [1]. It was first described in 1847 by Renaud and a little over 2000 cases have been reported to date. Its prevalence is believed to be higher than reported [2] because of its histological pattern (similar to epithelial ovarian cancer) and late stage diagnosis presenting with extensive pelvic dissemination (the ovary being considered the primary tumor). PFTC usually presents in the sixth decade of life [1] and the predisposing factors are not well determined, although nulliparity, infertility and inflammatory pelvic disease may have an influence [3]. The presence of *BRCA* genetic mutation confers an augmented risk of developing PFTC. Some suggest that in all PFTC cases encountered in their series 16% have *BRCA* mutations [4]. Symptoms include profuse vaginal discharge, abnormal vaginal bleeding, pelvic or abdominal pain (usually colicky pain due to tube distension) and a pelvic and/or abdominal mass can be palpable [5]. Latzke’s triad, which comprises watery vaginal discharge, vaginal bleeding with pelvic and/or abdominal pain, presents in only 15% of cases [6]. Preoperative diagnosis is difficult and imaging can be misleading because it may not identify the primary tumor. Pelvic ultrasound can be used to assess “sausage-like” images, related to tubal distension, as well as vascular abnormalities using Doppler ultrasound [7]. Depending on the series, there is a 3 to 4% rate of preoperative diagnosis [8]. Diagnosis is usually histological and has to meet specific criteria [9]. Tumor dissemination in cases of PFTC is preferentially transperitoneal and lymphatic [1] which can explain the higher incidence (in comparison to the ovary) of distant and retroperitoneal metastases [10]. Staging, surgical treatment and adjuvant medical treatment follow the same principles of epithelial ovarian cancers [11,12]. Prognosis is highly dependent of stage at diagnosis. A 5-year survival can range between 50 and 60% (stage II) and 10 and 20% (stages III and IV) [1]. Other series report different survival rates, but use more aggressive chemotherapeutic protocols. There are some unfavorable prognostic factors besides stage: age above 50-years old [13], tubal muscular layer (>50%) and/or serosal invasion [1], less optimal cytoreduction [12] and histological poor differentiation [14]. Surprisingly, primary tumor size does not affect prognosis [1].

## Case presentation

**Figure 1:**
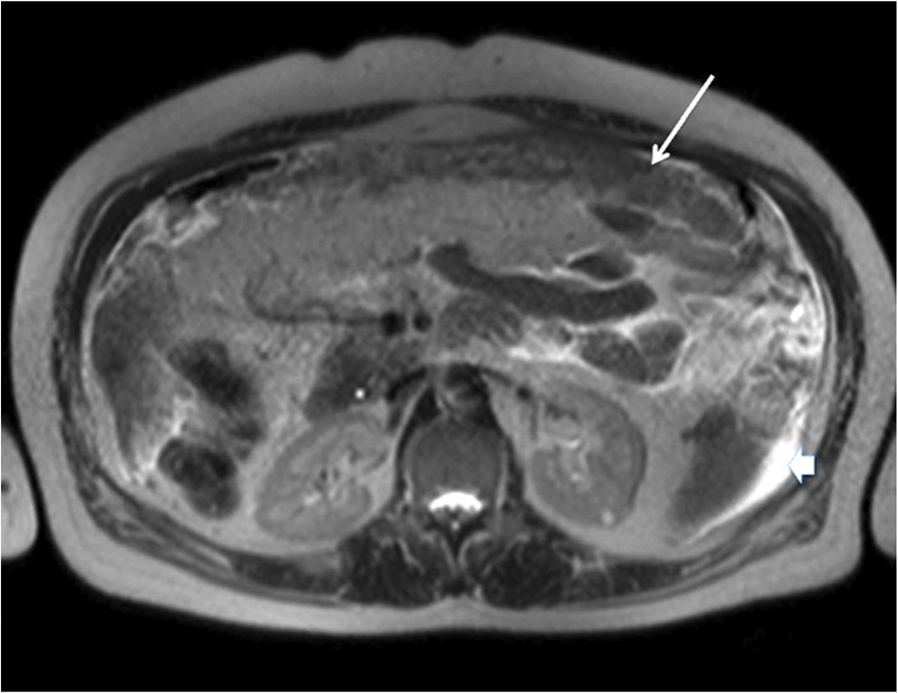
**Abdominopelvic magnetic resonance imaging.** Axial T2-weighted image. Heterogeneous soft-tissue thickening of the greater omentum (“omental cake”) (arrow). Moderate ascites (arrowhead).

This case report presents a 66-year-old Caucasian female housekeeper. Her background includes squamous carcinoma of the nose treated with surgery (14 years ago), hypertension and appendectomy (at a young age). She was nulliparous and had no previous known gynecological problems (menarche and menopause at normal ages, no combined hormonal contraceptives or hormonal replacement therapy). Her only symptom was diffuse abdominal pain, more intense on the flanks, progressing for 3 months and with increasing intensity. The family doctor requested a computed tomography scan that suggested peritoneal carcinomatosis, moderate ascites, with no reference to the primary tumor site. When further examined, a complete and thorough examination showed:

1. No vaginal discharge, endocervical polyp (no dysplasia) and normal cervical-vaginal cytology.

2. Blood work: elevation of CA-125 (515UI/mL), CA-19.9 and carcinoembryonic antigen within normal range, hepatic enzymes slightly elevated.

3. Diagnostic paracentesis: no malignant cells.

4. Gastrointestinal tract study: normal upper and lower endoscopy.

5. Abdominopelvic magnetic resonance imaging (MRI): moderate ascites, peritoneal thickening around transverse mesocolon and omental involvement (“omental cake”) (Figure 1) and a large, calcified uterine fibroid.

**Figure 2:**
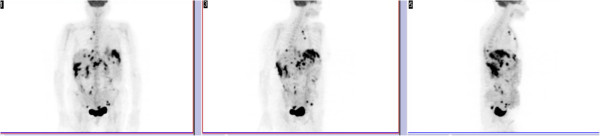
**Positron emission tomography scan.** Multiple ganglion metastases with several small implants on the pelvic and abdominal cavities (slides 1 and 3 – coronal section; slide 5 – sagittal section).

**Figure 3:**
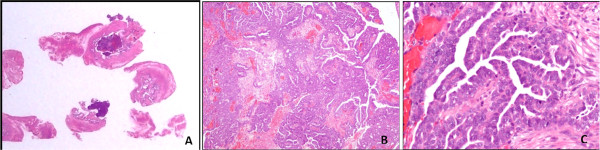
**Primary fallopian tube carcinoma.** Hematoxylin and eosin stain showing total inclusion of tubal neoplasia growing inside the lumen **(A)**; papillary serous adenocarcinoma with solid areas **(B)**; moderate nuclear atypia **(C)**.

The patient’s case was reviewed by the Gynecology team at the Portuguese Institute of Oncology of Oporto and an exploratory laparotomy was decided. The intra-operatory findings showed diffuse parietal peritoneal implants (pelvic and abdominal) with a voluminous mass involving her epiploon, from the hepatic hilum to the splenic hilum, invading the great gastric curvature. An anterior abdominal wall implant was removed and sent for extemporary examination which revealed high-grade adenocarcinoma. Ascitic fluid was collected; atypical gastrectomy, omentectomy and myomectomy were performed. When reviewing the adnexial area, a small dilation and purple color were noticed on her left fallopian tube. The surgery was completed with left adnexectomy. Macroscopic residual disease (>2cm) was present at the end of surgery. During the postoperatory period, a positron emission tomography scan revealed multiple ganglion metastases with several small implants on her pelvic and abdominal cavities (Figure 2). The pathology report showed infiltrative serous adenocarcinoma of epiploon and gastric curvature (papillary pattern Figures 3A and 3B), invading the gastric muscular layer, but not the mucosa. When analyzing her left fallopian tube, the same adenocarcinoma type was found in the lumen (9mm in size) but it had not invaded the muscular layer (Additional file 1: Figure S1 – A, B). Intraepithelial neoplastic segments were found in other parts of the tubal mucosa. Her left ovary was normal. Immunohistochemical analysis, using p53 and Ki-67 index, matched the tumor tissue in the abdominal implants with the tumor in her fallopian tube (Additional file 2: Figure S2), which led us to reach the conclusion of PFTC (higher differentiation of the tubal neoplasia and presence of intraepithelial neoplastic areas). The patient was then enrolled for six cycles of paclitaxel with carboplatin without complications.

## Discussion

The symptomatic presentation was very unspecific and the pelvic imaging found only a large uterine fibroid.

Optimal cytoreduction was not possible and hysterectomy with right adnexectomy was not considered because the primary tumor appeared to be gastric or peritoneal and residual upper abdominal tumor volume was high. The diagnosis of a PFTC was made with anatomical-pathological findings and immunohistochemistry tests, although the CA-125 descent pattern was also a compelling factor. The CA-125 measurement started at 518.5UI/mL, after surgery it was 59.94UI/mL and after two chemotherapy cycles the value was normal (13.13UI/mL).

Pelvic MRI is not totally specific for adnexal evaluation [15] and pelvic ultrasound with color Doppler could be helpful in better characterization of the pelvic organs [5].

The higher tubal neoplasia differentiation, the invasion only of her stomach’s outer layers, the presence of intraepithelial tubal neoplastic areas and the complete response to chemotherapy (imaging and the CA-125 descent), favors the diagnosis of PTFC with secondary peritoneal involvement. Extensive metastasis in the upper abdomen without PTFC muscle and serous coats breach is possible due to the exfoliation of malignant cells into the peritoneal fluid, followed by the natural flow of peritoneal fluid in the peritoneal cavity, which can take the metastasis to the upper abdomen.

## Conclusions

PFTC is a rare entity that poses diagnostic challenges. Symptoms, imaging and even pathological findings are not straight forward. In this case report we found a small PFTC (9mm) with extensive upper abdominal dissemination. This kind of presentation is unusual, although it presented at a late stage (International Federation of Gynecology and Obstetrics III) as do most PFTCs. The staging and adjuvant therapy in PFTC is similar to that of epithelial ovarian cancer and primary peritoneal cancer [11]. The apparent defiance of serous carcinomas to conform to the adenoma-carcinoma models is probably linked to the propensity of this tumor to spread early in its course. The dissemination process seems to be independent of initial primary tumor size and transperitoneal and lymphatic spread are more relevant [1]. In past years, peritoneal serous carcinogenic sequence has been reviewed in the light of new findings. Tubal fimbriae became the focus of the studies and the conclusions from these works are that the initial modified criteria by Hu *et al.*[16] for the diagnosis of PTFC detects only a portion of PTFCs and that the fallopian tube is a major site of origin for pelvic serous cancer irrespective of *BRCA* status.

Other recent studies from several cancer institutes have identified a number of promising biomarkers including HE4, mesothelin, and kallikreins. It would be possible to look for membrane and secreted proteins that distinguish the normal fallopian tube epithelium from its malignant counterpart. This is an essential step in the development of diagnostic and prognostic biomarkers for this disease.

## Consent

Written informed consent was obtained from the patient for publication of this case report and accompanying images. A copy of the written consent is available for review by the Editor-in-Chief of this journal.

## Competing interests

The authors declare that they have no competing interests.

## Authors’ contributions

CO reviewed the literature, analyzed the clinical case information and the imaging data and was a major contributor in writing the manuscript. DF contributed with surgical description, literature review and interpretation of patient data. HD provided the description of MRI slides and reviewed the final text. CB performed the histological examination and provided the description of the histological images. All authors read and approved the final manuscript.

## Supplementary Material

Additional file 1: Figure S1 Epiploon and gastric curvature. Hematoxylin and eosin stain showing infiltrative serous adenocarcinoma with papillary pattern (A). Severe nuclear atypia (B). Click here for file

Additional file 2: Figure S2 Immunohistochemical analysis. Immunohistochemistry stains showing intense and diffuse p53 signature on the affected mucosa (Fig. A) and high proliferative Ki67 index (Fig. B). Click here for file
