# Supplementary material for: Small fallopian tube carcinoma with extensive upper abdominal dissemination: a case report
Source: J Med Case Rep. 2013 Nov 7;7:252. doi: 10.1186/1752-1947-7-252 (PMC3835416; doi:10.1186/1752-1947-7-252)
Supplement: Additional file 1: Figure S1 — Epiploon and gastric curvature. Hematoxylin and eosin stain showing infiltrative serous adenocarcinoma with papillary pattern (A). Severe nuclear atypia (B). [file 1752-1947-7-252-S1.docx]

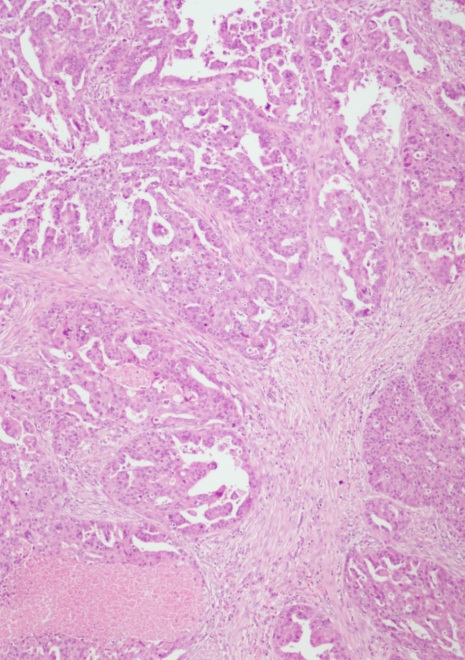


**A**


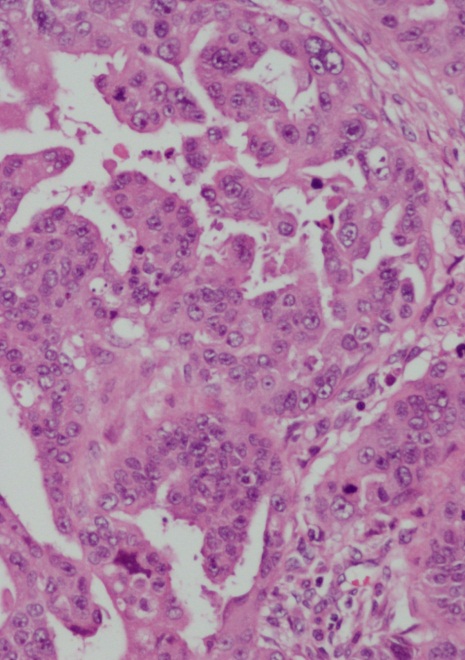


**B**

Additional file 1: Figure S1: **Epiplon and gastric curvature**

Hematoxylin and eosin stain showing infiltrative serous adenocarcinoma with papillary pattern (fig. A). Severe nuclear atypia (fig. B).
