# Supplementary material for: Small fallopian tube carcinoma with extensive upper abdominal dissemination: a case report
Source: J Med Case Rep. 2013 Nov 7;7:252. doi: 10.1186/1752-1947-7-252 (PMC3835416; doi:10.1186/1752-1947-7-252)
Supplement: Additional file 2: Figure S2 — Immunohistochemical analysis. Immunohistochemistry stains showing intense and diffuse p53 signature on the affected mucosa (Fig. A) and high proliferative Ki67 index (Fig. B). [file 1752-1947-7-252-S2.docx]

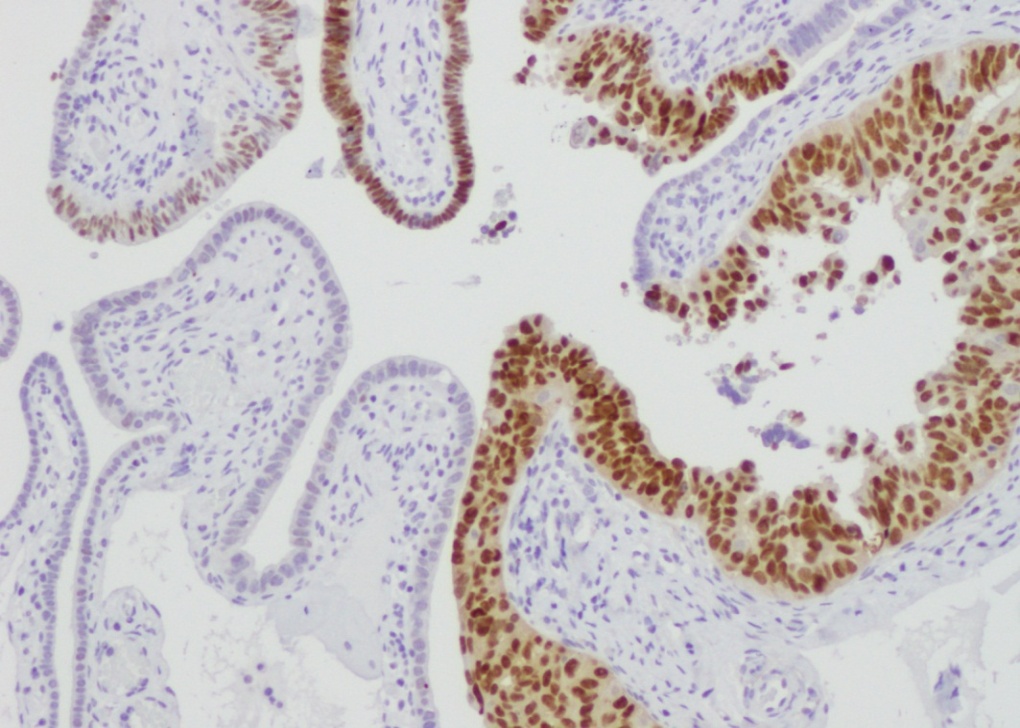


Fig. A

**p53**


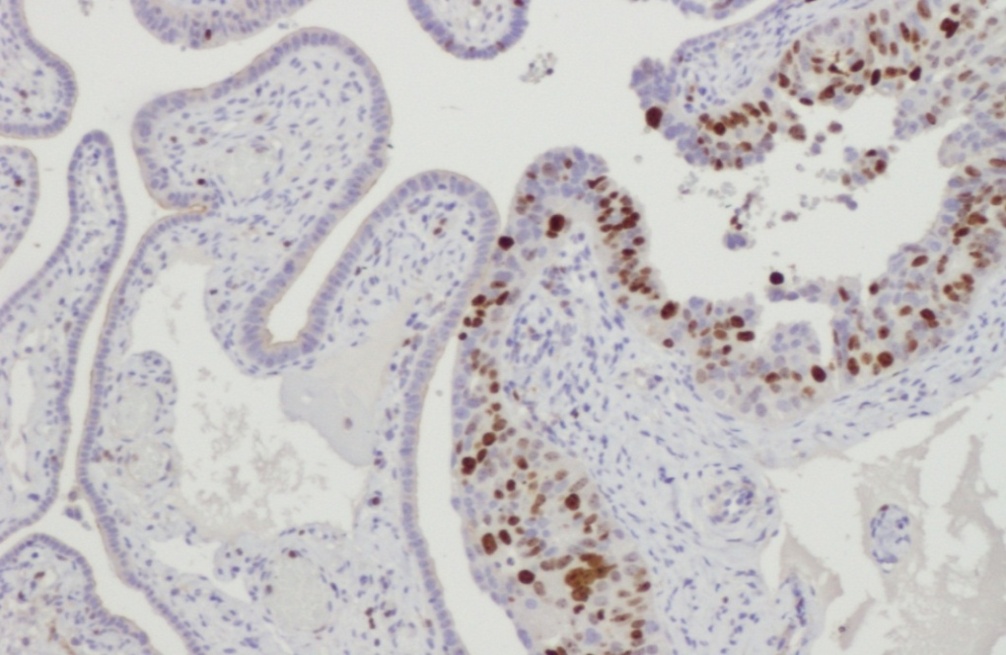


Fig. B

**Ki67**

Additional file 2: Figure S2: **Immunohistochemical analysis**

IHC stains showing intense and diffuse p53 signature on the affected mucosa (fig. A) and high proliferative Ki67 index (fig. B).
